# Clinical Efficacy of Vertical or Parallel Technique of a Micro‐Locking Plate for Treatment of Dubberley B‐Type Capitellar Fractures

**DOI:** 10.1111/os.12880

**Published:** 2021-01-10

**Authors:** Yao Lu, Lei Fu, Teng Ma, Yi‐bo Xu, Li‐ping Xu, Zhe Song, Shan Fan, Qian Wang, Liang Sun, Han‐zhong Xue, Zhong Li, Kun Zhang, De‐yin Liu, Cheng Ren

**Affiliations:** ^1^ Department of Orthopaedic Surgery Hong Hui Hospital, Xi'an Jiaotong University Xi'an China; ^2^ Bioinspired Engineering and Biomechanics Center (BEBC), The Key Laboratory of Biomedical Information Engineering of Ministry of Education School of Life Science and Technology, Xi'an Jiaotong University Xi'an China; ^3^ Orthopaedics Institute of Chinese PLA 80th Hospital Weifang China

**Keywords:** Capitellar fractures, Dubberley fracture, Fixation, Micro‐locking plate

## Abstract

**Objective:**

To evaluate the clinical efficacy of micro‐locking plate through vertical or parallel technique for treatment of Dubberley B‐type capitellar fractures.

**Methods:**

A retrospective analysis was performed in 24 patients (17 males and seven females, with an average age of 44.9 years, range from 19 to 75 years) with capitellar fractures that were treated with micro‐locking plate using vertical or parallel technique between January 2016 to January 2019. The inclusion criteria include closed capitellar fracture, normal anterior elbow joint movement before injury, and recent capitellar fracture with injury within past 3 weeks. Fractures classified according to Dubberley included four cases of type IB, eight cases of type IIB, and 12 cases of type IIIB. Radiographic evaluation was performed. Surgery time, blood loss, range of motion of the elbow, forearm rotation, and complications were recorded. Elbow joint function was evaluated by Mayo Elbow Performance Score (MEPS).

**Results:**

The mean follow‐up period was 19.6 months (range, 12–36 months). The average clinical healing time for fractures was 11.2 ± 3.2 weeks (range, 8–20 weeks). Fracture united in all patients. Two patients showed slight delayed union, but union was achieved eventually. The mean time from injury to surgery was 6.3 ± 3.1 days (range, 2–15 days). The average surgical time was 68.1 ± 11.5 min (range, 50–90 min), and the mean blood loss was 75.2 ± 26.5 mL (range, 40–120 mL). The mean range of flexion was 122.5° ± 10.5°(range, 95°–140°). The mean range of extension was 8.5° ± 5.8°(range, 0°–20°). The mean range of pronation was 79.7° ± 8.0°(range, 65°–90°). The mean range of supination was 80.5° ± 7.1°(range, 60°–90°). The mean MEPS at final follow‐up was 89.8 ± 9.0 (range, 60–100). Based on the MEPS, 18 (75%) patients had excellent, five (20.8%) patients had good, and one (4.2%) patient had fair. None of the 24 patients suffered vascular or nerve injury. One patient showed superficial infection, which was treated with surgical dressing.

**Conclusions:**

The vertical or parallel technique of the micro‐locking plate is an excellent method for treating Dubberley B‐type capitellar fractures.

## Graphical abstract

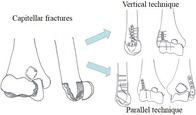

The micro‐locking plate accroding to vertical or parallel technique is an excellent method for Dubberley B‐type capitellar fractures.

## Introduction

Fracture of the capitulum of the humerus is a rare intra‐articular fracture of the distal humerus that comprises approximately 0.5% to 1% of elbow fractures^1^. The mechanism of injury typically includes falling on the extended arm, which causes direct axial pressure that is transmitted through the joint, leading to coronal shear fracture of the capitellum^1^. This type of fracture can easily be misdiagnosed by normal X‐ray examination due to the location of the fracture line on the frontal plane and the shear stress of the radial head acting on the capitulum^2^. In recent years, with the development of digital imaging and computed tomography, capitellar fractures can be accurately evaluated^3^. Currently, open reduction and internal fixation are the gold standard for treating this type of injury^4^. Bryan and Morrey classification has been widely used to classify capitellar fractures^5^. Type 1 (Hahn–Steinthal fracture) includes coronal shear fractures, involving a thick hemispherical fragment. Type 2 (Kocher–Lorenz fracture) is a cartilaginous “thin” fragment. Type 3 is comminuted and is multi‐fragmentary. Type 4 was added by McKee *et al*. to describe capitellar fractures that extend medially to involve most of the trochlea^6^. Recently, Dubberley *et al*. described a new classification system to guide surgical management and provide prognostic value^7^. Type 1 fracture of capitellum involves the capitellum with or without the lateral trochlea ridge. Type 2 fracture involves a single fragment of capitellum and trochlea. Type 3 fracture involves separate fragments of capitellum and trochlea. Each type is further classified as either A (no posterior comminution) or B (posterior comminution).

At present, various treatment methods have been proposed for such fractures. But there is not a final conclusion. Such fractures present insufficient bone for internal fixation, making surgical treatment difficult^2^, ^8^. Several studies have recommended Kirschner wires and bio‐absorbable screws for capitellar fractures^8^, ^9^. However, Kirschner wires and bio‐absorbable screws fail to provide sufficient fixation strength, leading to less favorable results. Most studies recommend that intact bone fractures can be generally treated by using a screw for fixation^10^, ^11^. Ruchelsman^12^ reported that 16 skeletally mature patients with a closed capitellar fracture were treated with buried cannulated variable‐pitch headless compression screws through an extensile lateral exposure. The mean Mayo Elbow Performance Index score was 92 ± 10 points, with nine excellent results, six good results, and one fair result. A randomized controlled trial conducted by Yu^13^ compared the Herbert screw fixation between the lateral approach and the anterolateral approach in 26 patients and demonstrated that both lateral approach and anterolateral approach with Herbert screw internal fixation are suitable for coronal shear fractures of capitellum with satisfactory early outcomes. However, screw internal fixation is not suitable in cases of Dubberley B‐type fractures involving incomplete capitulum fracture of the humerus behind the lateral condyle^7^. Because the fracture fragments provides a limited channel for the screw position in such cases, screw fixation is ineffective.

Therefore, there is still an unmet need for developing a suitable alternative fixation approach for this type of fracture. Previously, we tested the use of a micro‐plate and single‐plane fixation^14^. In subsequent follow‐up, however, cases were found to have internal fixation failure and fracture displacement. Therefore, we presented a technique for the internal fixation of capitellar fractures using the micro‐locking plate with vertical or parallel techniques. We use generic questionnaires to evaluate outcome scores of surgical techniques. The Mayo Elbow Performance Score (MEPS) is a widely used measuring index to evaluate clinical outcomes for a variety of elbow disorders, which was introduced in the year 1985 by Morrey *et al.* ^15^. This scoring system was modified to evaluate the results of treatment of elbow fractures and dislocations by Broberg and Morrey^15^. It consists of assessment of arc of motion, stability, pain, and a patient rating of daily function.

In this study, patients with capitellar fractures that were treated with vertical or parallel locking plate techniques were retrospectively reviewed. The purpose of this study was as follows. First, we intended to describe vertical or parallel locking plate strategy for the treatment of capitellar fractures. Second, we aimed to evaluate the effificacy and feasibility of vertical or parallel locking plate techniques in treating capitellar fractures. Third, we tried to provide more evidence to guide the management of capitellar fractures based on the surgical skills and our significant results.

## Patients and Methods

### *Inclusion and Exclusion Criteria*

The inclusion criteria were as follows: (i) diagnosis of closed capitellar fracture with normal anterior elbow joint movement before injury; (ii) vertical‐ or parallel‐locking plate techniques for the treatment of capitellar fracture; (iii) postoperative follow‐up ≥12 months; and (iv) retrospective study. Exclusion criteria were as follows: (i) pathological fracture; (ii) severe osteoporosis; (iii) those who could not be contacted; (iv) individuals who refused follow‐up; and (v) those with incomplete clinical data before and/or after surgery.

### *Patient Data*

A retrospective study reviewed a consecutive series of 24 patients with capitellar fractures who presented at our center between January 2016 and January 2019. Plain radiographs and computed tomography (CT) scans were obtained immediately after the injury, and were reviewed by two experienced orthopaedic surgeons. Patient demographic characteristics, including age, gender, side of injury, mechanism of injury, type of fracture (Dubberley classification system^7^), time from injury to surgery, and fixed technology, are shown in Table 1. The study was reviewed and approved by the Ethics Committee of Honghui Hospital, Xi'an Jiaotong University. All patients provided signed informed consent.

**TABLE 1:** Summary of the patient variables and outcomes of capitellar fractures

Patient	Age	Sex	Side	Mode of	Type of	Time from injury	Fixed	surgery time	blood lose	Time for fracture	follow‐up	Extension	Flexion	Pronation	Supination	MEPS	Complications
No.	(years)	(M/F)	(L/R)	injury	fracture	to surgery (d)	technology	(min)	(mL)	union(weeks)	(month)	(°)	(°)	(°)	(°)	score	
1	19	M	L	Fall	IIIB	9	vertical	65	50	8	36	0	140	90	90	100	None
2	25	M	L	Fall	IIB	8	parallel	80	60	12	24	10	130	70	80	85	None
3	36	M	R	Fall	IIB	4	parallel	75	45	8	12	15	125	85	80	95	None
4	45	F	R	Fall	IIIB	8	vertical	75	100	12	12	5	125	85	90	90	None
5	75	F	R	Fall	IB	2	vertical	60	120	20	14	15	120	75	85	90	Delayed union
6	60	M	L	Fall	IIB	12	vertical	90	110	12	25	20	95	70	80	60	None
7	30	M	L	Falls from a height	IIB	5	parallel	65	100	12	16	10	115	90	85	95	None
8	36	M	L	Traffic accident	IIIB	5	parallel	50	95	8	36	5	125	85	80	95	None
9	52	M	L	Traffic accident	IIIB	4	vertical	55	40	12	12	5	125	90	85	100	None
10	55	M	R	Fall	IB	8	parallel	65	45	8	16	20	115	65	70	80	None
11	43	F	L	Fall	IB	10	parallel	70	90	8	18	15	100	70	75	75	None
12	40	M	L	Falls from a height	IIIB	4	vertical	70	60	13	23	5	135	80	80	95	None
13	40	M	R	Falls from a height	IIIB	7	vertical	85	100	8	13	10	120	88	80	95	None
14	50	M	R	Fall	IB	3	vertical	55	50	9	25	10	135	85	85	100	None
15	62	M	R	Traffic accident	IIB	3	vertical	60	40	12	24	5	120	80	90	95	Superficial infection
16	65	F	L	Traffic accident	IIIB	4	vertical	60	50	12	12	15	130	85	85	90	None
17	52	F	L	Fall	IIIB	6	vertical	65	60	16	12	10	115	70	75	85	None
18	36	F	R	Fall	IIIB	8	vertical	55	70	8	12	5	125	65	60	90	None
19	32	M	R	Falls from a height	IIIB	6	vertical	85	90	10	24	10	130	84	76	95	None
20	40	M	L	Fall	IIIB	6	parallel	55	80	9	18	5	130	85	85	90	None
21	41	M	L	Falls from a height	IIB	5	parallel	90	120	11	30	0	125	75	85	90	None
22	56	M	L	Traffic accident	IIB	5	vertical	70	100	18	28	5	110	75	70	80	Delayed union
23	59	F	L	Traffic accident	IIB	4	vertical	65	80	12	16	5	120	85	75	90	None
24	28	M	R	Falls from a height	IIIB	15	vertical	70	50	10	12	0	130	80	85	95	None

### *Surgical Strategy*

#### *Operative Position and Anesthesia*

**Fig 1:**
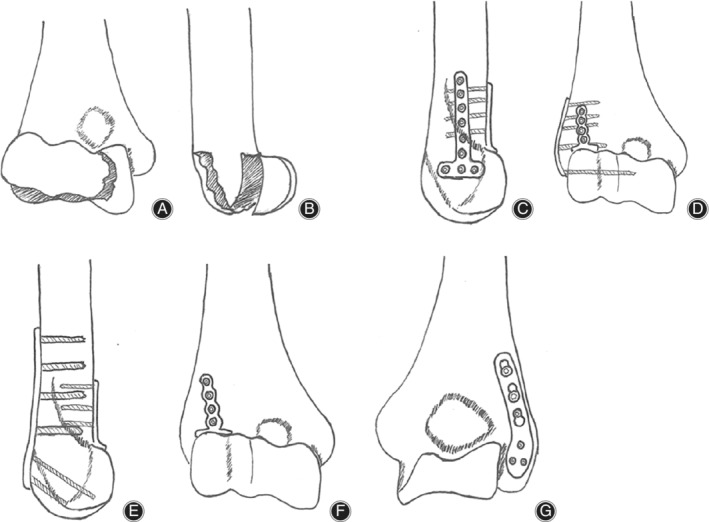
Preoperative planning. Dubberley IIB type: (A) lateral view; (B) AP view. (C, D) Micro‐locking plate vertical technique. (E–G) Micro‐locking plate parallel technique.

Preoperative planning shows in Fig. 1. The patient was situated in a supine position with the affected limb positioned alongside the body or to the side of the operating table after general or brachial plexus anesthesia.

#### *Incision and Exposure*

**Fig 2:**
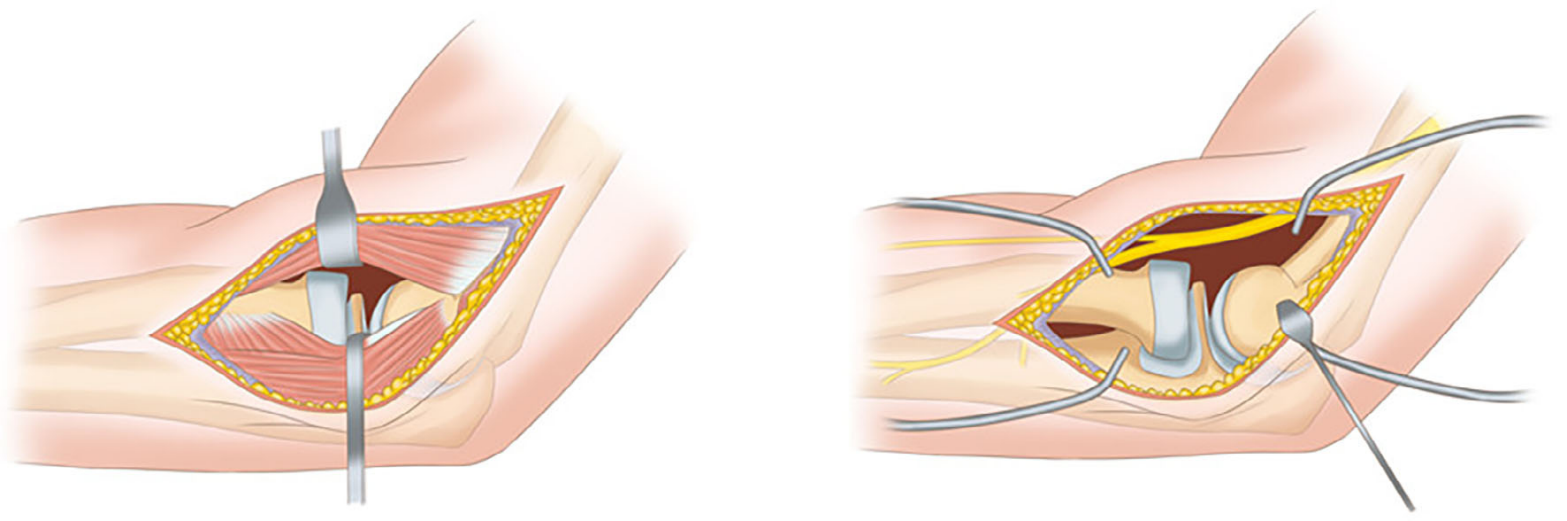
Kocher approach.

The stability of the elbow joint was checked. The classic lateral Kocher approach was used in all cases (Fig. 2). An incision was made from the lateral to the posterolateral side of the distal humerus, 1 to 2 cm below the capitulum of humerus and 5 to 7 cm above the articular surface of the proximal elbow joint. After exposing the lateral side of the distal humerus, the extensor and the full layer of the articular capsule in front of the elbow joint were reversed from the lateral side to the medial side of the distal humerus. The interosseous nerve is usually not affected and therefore does not require dissection. The elbow joint was flexed 30° to 45° and a medium Hohmann hook was inserted into the anterior articular capsule below the medial column of the humerus. This did not involve release of the radial collateral ligament. Soft tissue and hematoma filling the capitulum fracture and trochlear of the humerus were removed.

#### *Fixation*

**Fig 3:**
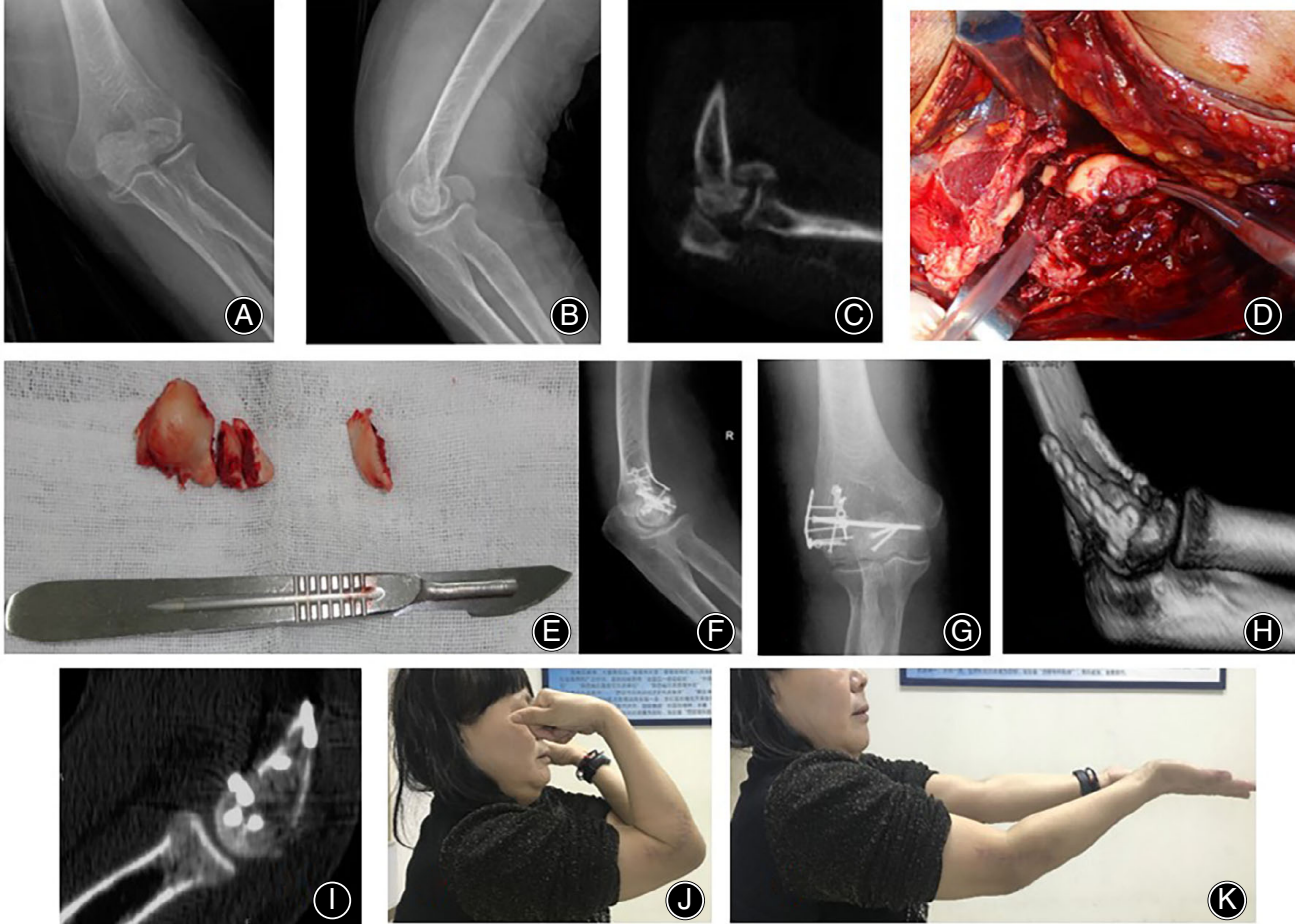
A 52‐year‐old female patient with capitellar fractures (Dubberley IIIB type) caused by fall from a height. Patient was treated with opening and micro‐locking plate fixation 10 days after injury. (A, B) Preoperative anteroposterior and lateral X‐ray examination showing frontal plane fracture of the distal end of the humerus; (C) Preoperative CT scans showing capitulum and trochlear fracture of the humerus; (D) Dissect and expose the fracture site; (E) The fragment is comminuted and dissociative sometimes; (F, G) Anteroposterior and lateral X‐ray is used to determine the fracture reduction and the placement of implants; (H, I) CT scans show satisfactory reduction and internal fixation; (J, K) functional appearance 1 year after operation showing satisfactory elbow function.

**Fig 4:**
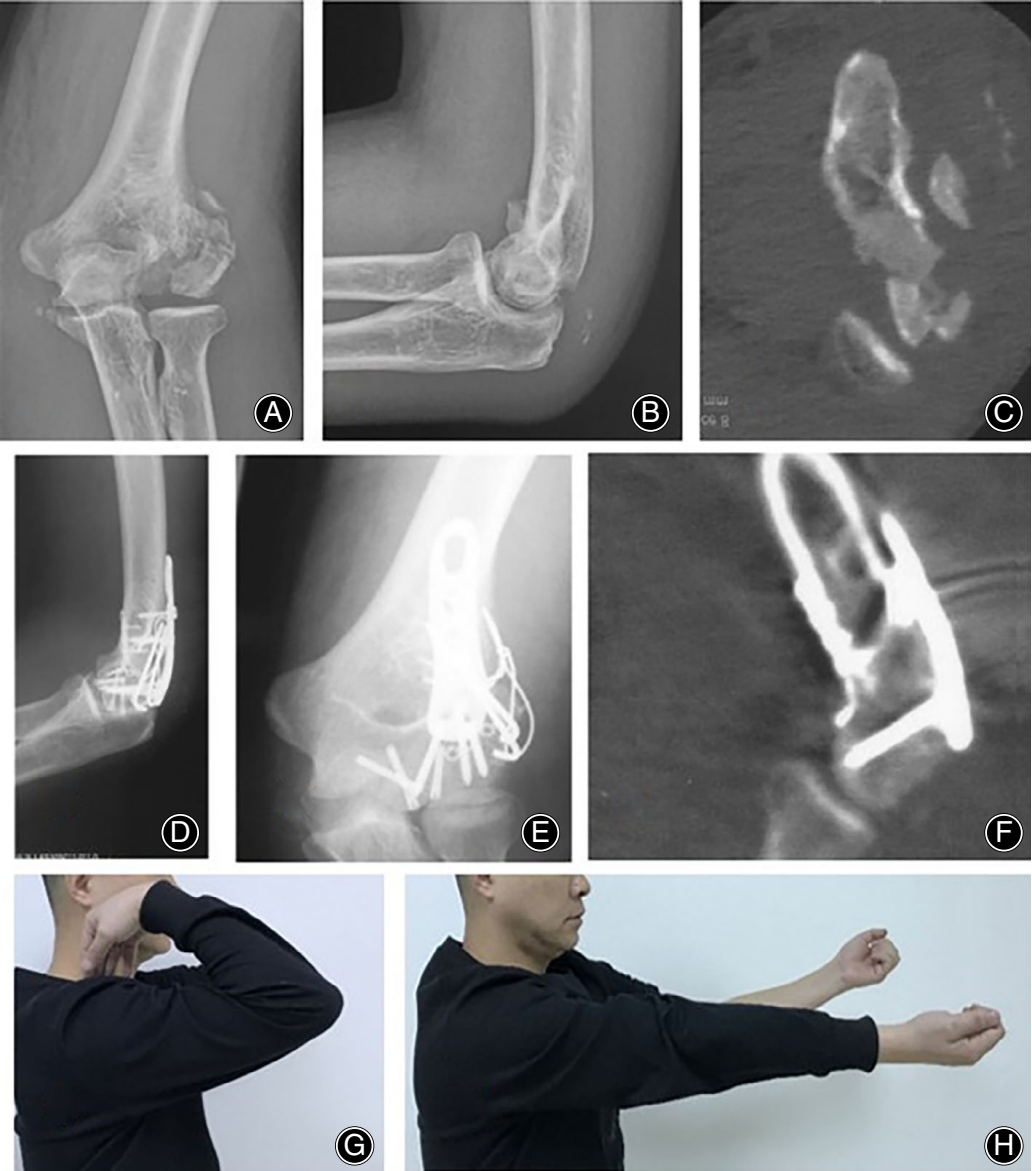
A 36‐year‐old male patient with capitellar fractures (Dubberley IIIB type) and avulsion fracture of the triceps brachii junction. Patient was treated with opening and micro‐locking plate parallel fixation 6 days after injury. (A–C) Preoperative anteroposterior and lateral X‐ray and CT scan showing frontal plane fracture of humerus. (D–F) CT and X‐ray scans 2 days after surgery showing satisfactory reduction and internal fixation. (G, H) Satisfactory recovery of elbow function 1 year after operation.

After reduction of fractures with point reduction forceps, a 1.0 mm Kirschner wire or 2.0 mm screw (Tianjin Zhengtian Medical Instrument Co., Ltd.) was used to fix the distal humeral joint from outside to inside. The reduction was confirmed visually and radiographically. A “T” shaped locking micro‐plate (Tianjin Zhengtian Medical Instrument Co., Ltd.) was fixed to the top of the articular surface of the capitulum of the humerus. The plate was positioned on the lateral side of the humeral trochlea close to the top of the coronary sulcus at the junction of the articular surface of the capitulum of humerus. Another “T” locking micro‐plate is attached to the lateral (Fig. 3) or posterior (Fig. 4) side of the lateral condyle of the humerus to support and fix capitellum. Elbow flexion was monitored during the operation to ensure absence of abnormal activity, blockage, or friction. All patients underwent intraoperative C‐arm X‐ray fluoroscopy to confirm that the fracture was properly repaired and the screw length was correct. The wound was flushed with isotonic saline and elbow joint flexion and extension functions were checked to ensure that excessive internal fixation would not result in movement blockage. Ulnar collateral ligament was repaired if damage was indicated by unstable valgus stress on the elbow. The surgical site was completely drained and the wound was sutured layer by layer. The elbow joint braced at an angle of 90°.

### *Postoperative Management*

Antibiotics were administered 30 min before and 24 to 48 h after surgery. Oral indomethacin was administered starting on the 2nd day post‐surgery to prevent heterotopic ossification. The elbow joint brace maintaining 90° elbow flexion lock was released 2 to 3 days after surgery to permit active and passive extension and flexion activity from 60° to 110°. Range of motion was increased gradually after 2 weeks. Rehabilitation exercise for forearm rotation function was then started. Fracture position and healing were checked by X‐ray examination at 4 weeks. Elbow joint extension and flexion range were increased and rehabilitation exercises for forearm rotation function were enhanced. Following confirmation of fracture healing at 8 to 12 weeks post‐surgery, upper limb weight‐recovery function training was gradually introduced.

### *Outcome Measures*

Patient general results, including age, gender, side of injury, mechanism of injury, type of fracture, time from injury to surgery, fixed technology, blood loss, surgical time, followed‐up time, clinical healing time, complications, and range of motion of the elbow and forearm rotation, were analyzed.

#### *The Mayo Elbow Performance Score (MEPS)*

The MEPS was used to evaluate postoperative recovery of elbow function in an adult population. The MEPS score system mainly includes four aspects: arc of motion, stability, pain, and a patient rating of daily function. The score standard had a maximum of 100 points (best possible outcome). A total score <60 is considered a poor score, 60–74 fair, 75–89 is good, and 90–100 excellent.

### *Statistical Analysis*

Statistical data were processed using GraphPad Prism7.0. Data were expressed as mean ± standard deviation. Comparison between two groups was performed using independent sample student's t‐test. Categorical data was performed using χ^2^ test. *P* < 0.05 was considered statistically significant.

## Results

### *General Results*

There were 24 patients with an average age of 44.9 years (range, 19–75 years) in this study. All fractures were closed. According to the Dubberley classification system, there were four patients with type IB fractures, eight patients with type IIB fractures, and 12 patients with type IIIB fractures. The mean time from injury to surgery was6.3 ± 3.1 days (range, 2–15 days). The average surgical time was 68.1 ± 11.5 min (range, 50–90 min). The mean blood loss was 75.2 ± 26.5 mL (range, 40–120 mL) (Table 1).

### *Follow‐up*

The patients were followed up after operation by questionnaire survey, medical history review, and outpatient follow‐up. The mean follow‐up time was 19.6 ± 7.7 months (range, 12–36 months).

### *Radiographic Improvement*

There were 18 cases exhibiting anatomical fracture reduction and six cases with functional reduction (less than 2 mm displaced) according to the radiographic review.

### *Clinical Improvement*

The average clinical healing time for fractures was 11.2 ± 3.2 weeks (range, 8–20 weeks). Fracture united in all patients although two patients showed slight delay in union (fracture united after 18 weeks) **(**Table 1 **).**

### *Functional Evaluation*

Range of motion of the elbow and forearm rotation.

The mean range of flexion was 122.5° ± 10.5° (range, 95°–140°). The mean range of extension was 8.5° ± 5.8° (range, 0°–20°). The mean range of pronation was 79.7° ± 8.0° (range, 65°–90°). The mean range of supination was 80.5° ± 7.1° (range, 60°–90°) (Table 1).

### *MEPS*

The mean MEPS at final follow‐up was 89.8 ± 9.0 (range, 60–100). Based on the MEPS, 18 (75%) patients had excellent, five (20.8%) patients had good, and one (4.2%) patient had fair (Table 1). Four aspects as arc of MEPS showed in Supplementary material 1.

#### *Complications*

None of the 24 patients suffered vascular or nerve injury. One patient showed superficial infection, which was treated with surgical dressing. No instability was observed in the medial stress test (Table 1).

## Discussion

The capitulum is located on the lateral side of the distal humerus and protrudes forward and downward. It functions largely to maintain the stability of the elbow joint^16^, ^17^. Capitellar fracture, with or without humeral trochlea fracture, is intra‐bone. Most displaced fracture blocks have no obvious soft tissue attachment and cannot be reset by ligament reduction techniques. It is generally accepted that surgical treatment is superior to non‐surgical treatment with respect to a number of clinical outcomes^18^, ^19^.

During surgery, the comminuted small bone can and should be used to reset and fix the fracture rather than dissecting and discarding it. However, when fixation is unreliable it should be removed in order to avoid mechanical blockage of joint activity. Small bones and associated soft tissues such as articular capsules feature good blood supply, and post surgery they participate in bone repair and accelerate fracture healing. Conversely, a small damaged fracture block exposes the fracture surface directly to the articular cavity, which can lead to traumatic arthritis, ossifying myositis, and even joint instability that eventually can severely affect elbow joint function^19^. Ashwood *et al*. considered it very important to maintain firmness following fracture reduction, thus small cartilage blocks of the elbow joint should be maintained as required for reduction and internal fixation during surgery^20^. Jupiter *et al*. reported that elbow joint function at the humeral distal frontal plane was correlated with the recovery of normal anatomy^21^. In the current study, 24 patients with capitellar fractures were treated by vertical or parallel micro‐locking plate technique. We found some advantages of this approach, including stable fixation, early resumption of elbow joint activity, and good functional recovery.

### *Selection of Surgical Approach to Capitellar Fracture*

There is currently no uniform, standard guide to selecting the optimal surgical approach to treating capitular fractures. Singh *et al*. describes surgeries including the anterior approach to the elbow and the posterior approach to the ulnar olecranon^22^. However, the classic posterior lateral Kocher approach is used for treating most distal articular surface fractures of the humerus. In our view, the choice of surgical approach depends largely on the shape of the fracture, the direction of displacement and the surgeon's familiarity with a given approach. The anterior approach of the elbow joint is complicated and includes risk of damage to blood vessels and nerves^23^. The posterior approach of the ulnar olecranon can reveal posterior condyle fracture but entails more surgical trauma and increased probability of heterotopic ossification in the elbow joint^21^. The posterolateral Kocher approach of the elbow joint provides good exposure, relatively decreased trauma, increased safety, and fewer postoperative complications^23^, ^24^. Sano *et al*. reported good clinical results using a lateral approach for fracture reduction and internal fixation in patients with distal humeral frontal plane fractures^25^. For all 24 patients in the current study, the classic lateral Kocher approach resulted in good exposure, reduction, and fixation. No other surgical incisions were made and no obvious elbow instability was observed.

### *Assessing Dubberley B‐Type Fracture Treatment Outcomes*

It is difficult to compare clinical results of different treatment methods for capitellar fractures, largely due to low incidence. However, additional factors also contribute to the difficulty of assessing outcomes. Young patients often suffer high‐energy injuries that usually feature combined composite injury of elbow joint structure^23^. Due to severe crushing of the fracture the joint remains unstable after simple bone structure repair. In elderly patients, most often with low‐energy injuries, fractures are not severely crushed but local compression and poor bone condition result in loosening of internal fixation and displacement of fractures^1^. If the small bones of the articular surface are preserved during fracture comminution, internal fixation is difficult and the fracture block is easily loosened after surgery^17^. It becomes a block in the joint that affects activity. If, however, the small bones of the articular surface are not preserved. the shape of the ankle joint and the ulnar joint surface will be altered and the humeroradial and ulnar joints will not match. This will result in an unstable elbow joint and lead to traumatic osteoarthritis^17^. In our view, surgery to treat capitellar fractures with or without trochlear fracture should aim to restore a uniform match of the humeroradial and humeroulnar joint, strongly fix the fracture, maintain fixation and joint stability, and restore maximum joint activity range and function. Achieving these aims depends critically on choosing the appropriate approach to internal fixation.

### *Fixation Method and Positioning*

Independent screw fixation is commonly used for internal fixation and is associated with good outcomes in previous studies^22^, ^26^, ^27^. However, it is an appropriate technique for simple fractures, such as no bone loss present or posterior comminution (Dubberley Type 1A and 2A)^27^. Sano *et al*. report that in the case of a capitulum of the humerus fracture with a thin fracture block, the screw thread will not fully pass the fracture line and function as a lag screw if inserted from the rear. If the fracture block is too small, the screw may damage the joint surface or cause the bone to split. Additionally, if the fracture block is too small it is difficult to seat the screw thread in the cartilage^25^. It has also been certified that the screw will damage the articular cartilage leading to cartilage necrosis or osteolysis and affect elbow joint function^20^. However, this ensures only the stability and firmness of the frontal plane but not the effective fixation of a comminuted posterior condyle or incomplete humeral external condyle fracture that would permit early functional rehabilitation exercises. Studies involving greater numbers of patients and extended follow‐up show that distal frontal plane and anti‐sliding plate treatments do not guarantee stability and firmness of frontal plane fractures nor ability to perform early functional exercises for patients with osteoporosis^28^. Therefore, a suitable approach to enchance the stability of fixation remained uncertain. So, we put forward vertical and parallel techniques. First, we used a Kirschner wire and screw to fix the fracture. Then, a micro‐locking plate was placed on the posterior side of the humerus for support and fixation of the lateral and posterior humerus. Finally, a micro‐locking plate was used in the front of fracture for anti‐glide. Our technique has several advantages over previous methods of fixation. Firstly, the 1.5 mm and 2.0 mm micro‐locking system screws can meet the requirement for multiple screws on the fracture block, and the small screws can replace the Kirschner wire. Secondly, the locking‐plate screw‐fracture block can be completely integrated into one body in which loosening of the screw and bone plate breakage are unlikely^29^. The advantage of this approach is particularly apparent for patients with comminuted fracture or osteoporosis, requiring support and fixation that restores the original length. Thirdly, two‐plane internal fixation of the distal end of the humerus using a micro‐locking plate not only achieves front and side anti‐slip and lateral and posterior support, but also effectively covers a crushed fracture, thereby guaranteeing stability and firmness and also maintaining stability and compatibility of the articular surface after reduction. It affords maximal fixation stability that promotes early rehabilitation of elbow joint through active and passive flexion and extension exercises to fully restore elbow joint function. The minimal amounts of built‐in material reduce irritants otherwise adversely affecting later functional exercises. Follow‐up of the 24 patients in this study showed stable internal fixation, no displacement, and good position of the fracture. All of the patients displayed early recovery, with satisfactory elbow function.

### *Limitations*

The study was limited by the small number of cases (*n* = 24), some bias in patient selection, short follow‐up period, and lack of biomechanical studies. Whether or not this approach can be widely applied in the clinic must be further determined in larger studies enrolling greater numbers of patients with long‐term follow‐up and biomechanical assessments.

## Conclusion

Selection of a surgical treatment approach to Dubberley B‐type fracture of the capitulum of the humerus requires CT scan examination to determine the extent and degree of comminution of the fracture. The classic Kocher approach reveals the full range of the fracture. Following fracture reduction, use of a micro‐locking plate with vertical or parallel technique resulted in early performance of functional exercises and did not yield obvious postoperative complications.

## Supporting information


**Appendix S1**. Supplementary material.
