# Supplementary material for: Clinical Efficacy of Vertical or Parallel Technique of a Micro‐Locking Plate for Treatment of Dubberley B‐Type Capitellar Fractures
Source: Orthop Surg. 2021 Jan 10;13(1):207–15. doi: 10.1111/os.12880 (PMC7862171; doi:10.1111/os.12880)

**supplemental material 1**

Four aspects as arc of MEPS

| Patient | MEPS (score) | | | | Total |
| --- | --- | --- | --- | --- | --- |
| No. | Pain | motion | stability | daily function |
| 1 | 45 | 20 | 10 | 25 | 100 |
| 2 | 30 | 20 | 10 | 25 | 85 |
| 3 | 45 | 20 | 10 | 20 | 95 |
| 4 | 45 | 20 | 10 | 15 | 90 |
| 5 | 45 | 20 | 10 | 15 | 90 |
| 6 | 30 | 15 | 10 | 5 | 60 |
| 7 | 45 | 20 | 10 | 20 | 95 |
| 8 | 45 | 20 | 10 | 20 | 95 |
| 9 | 45 | 20 | 10 | 25 | 100 |
| 10 | 30 | 15 | 10 | 25 | 80 |
| 11 | 30 | 15 | 10 | 20 | 75 |
| 12 | 45 | 20 | 10 | 20 | 95 |
| 13 | 45 | 20 | 10 | 20 | 95 |
| 14 | 45 | 20 | 10 | 25 | 100 |
| 15 | 45 | 20 | 10 | 20 | 95 |
| 16 | 45 | 20 | 10 | 15 | 90 |
| 17 | 30 | 20 | 10 | 25 | 85 |
| 18 | 45 | 20 | 10 | 15 | 90 |
| 19 | 45 | 20 | 10 | 20 | 95 |
| 20 | 45 | 20 | 5 | 20 | 90 |
| 21 | 45 | 20 | 10 | 15 | 90 |
| 22 | 30 | 20 | 10 | 20 | 80 |
| 23 | 45 | 20 | 10 | 15 | 90 |
| 24 | 45 | 20 | 10 | 20 | 95 |

**supplemental material 2**

A 51 year-old female patient with capitellar fractures (Dubberley IIIB type) caused by fall from a height. Patient was treated with micro-locking plate fixation. A, B. Preoperative anteroposterior and lateral X-ray examination showing capitellar fractures ; C,D. Preoperative CT scans showing capitulum and trochlear fracture of the humerus; E,F.CT scans showing satisfactory reduction and internal fixation of capitellar fractures and good recovery of distal articular surface. G,H. Anterior and lateral X-ray films show fracture union at 1year after operation.


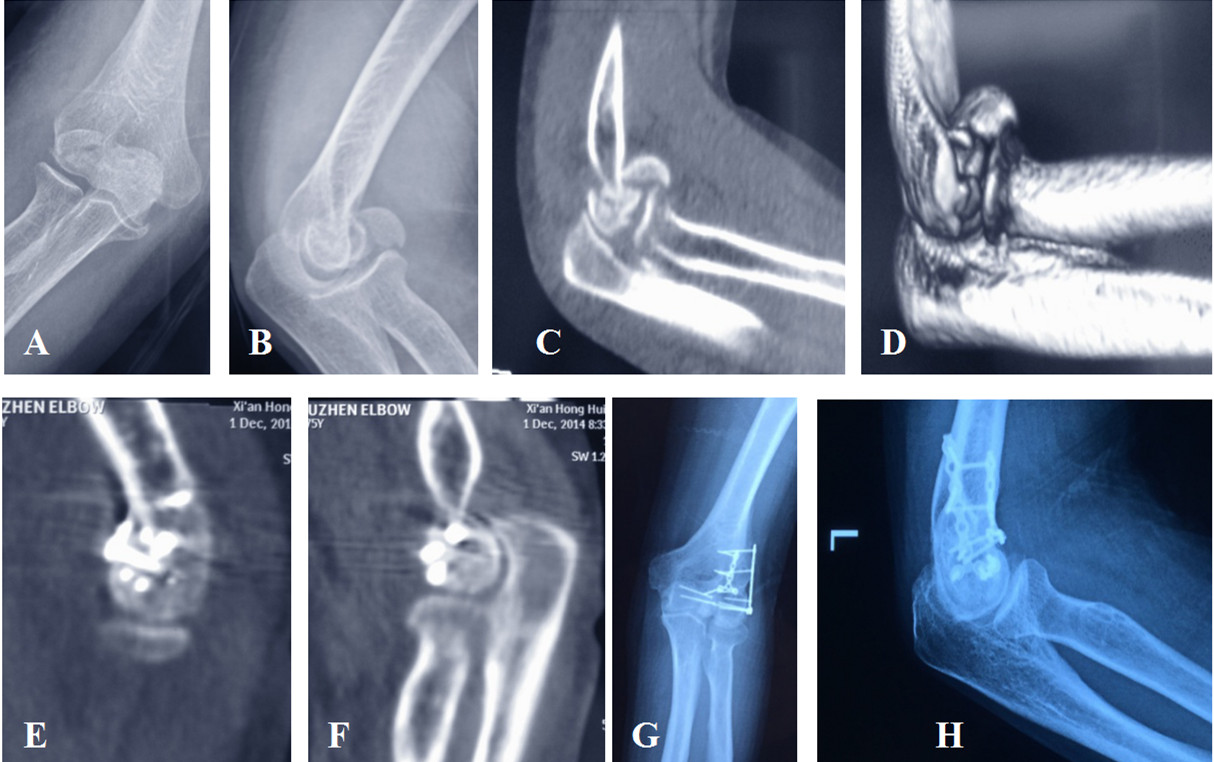

Supplement: Supplementary file 1 — Appendix S1. Supplementary material. [file OS-13-207-s001.doc]
